# The political economy of reshoring: Evidence from the semiconductor industry

**DOI:** 10.1371/journal.pone.0316473

**Published:** 2025-02-14

**Authors:** Richard Clark, Sarah Kreps, Adi Rao

**Affiliations:** 1 University of Notre Dame, Notre Dame, IN, United States of America; 2 Cornell University, Ithaca, NY, United States of America; Thammasat University Institute of East Asian Studies, THAILAND

## Abstract

States have increasingly taken steps to reshore manufacturing by shifting production back home from abroad. Although the literature on responses to globalization is increasingly robust, scholars tend to focus on the political consequences of economic dislocations, the effectiveness of government policies intended to roll back globalization, and the macroeconomic consequences of such policies. Our research is among the first to study public attitudes toward reshoring, including the basis of support, demographic variation, and sensitivity to factors like consumer prices. We advance hypotheses about the role of national security considerations and economic self-interest in shaping why and under what conditions the public backs incentives and investments that bring manufacturing home. We field two pre-registered survey experiments to representative samples of the American public, finding that US citizens support reshoring in most cases, irrespective of political party. Only the prospect of severe price increases and reshoring from geographically proximate allies moderate these responses. Our findings advance literatures on reshoring and public attitudes toward economic relationships, with implications for policy as governments consider reshoring a range of industries from technology to pharmaceuticals.

## Introduction

Reshoring, defined as “the decision to bring back to the home country production activities earlier offshored,” has been on the rise in the United States over the last decade [1, 79]. The Reshoring Initiative reports that a total of 1.3 million manufacturing jobs have been brought back to the United States since 2010 [2]. Although globalization raised global economic output, economists and policymakers have also observed distributional consequences in the form of lost manufacturing jobs and reduced wages that have prompted aggressive policies intended to reverse the trend of moving manufacturing to locations with lower labor costs [3, 4].

These policies, many of which had their roots in the Trump administration’s tariffs that made imports more expensive and aimed to level the manufacturing playing field, gained additional momentum in 2021 as the pandemic triggered shortages in supply chains. Reshoring became a way to promote resilience, circumventing pandemic labor restrictions abroad, logistics challenges, and obstacles at domestic ports.

While several industries experienced supply chain disruptions, nowhere was the issue as acute and salient as in semiconductors. Semiconductor chips are essential components in nearly every piece of modern electronics, from smartphones and computers to cars, medical devices, and military equipment. They play a vital role in cutting-edge technologies like artificial intelligence, 5G, and defense systems. Countries thus view semiconductor manufacturing as a strategic asset for national security. Any interruption can compromise critical infrastructure, national defense, and technological leadership. Over the last several decades, the United States has steadily lost market share in semiconductor manufacturing, from 37 percent in 1990 to 12 percent in 2021. The United States Secretary of Commerce Gina Raimondo acknowledged that the US no longer produces any of the most advanced semiconductor chips [5, 6].

Policymakers argued that if semiconductor chips were produced at home, the United States would be more resilient in the face of supply chain shocks and more competitive geopolitically [7]. They also argued that reshoring was a way to redress longer-run job losses in industries like steel, a buffer against decarbonization-fueled unemployment [8, 9], and insurance against a potential Chinese invasion of Taiwan [10]. To that end, the Chips and Science Act passed the Senate in July 2022 with a lopsided bipartisan vote; the bill led some observers to conclude that “semiconductor shortages end an era of globalization” [11].

The framing of reshoring as a way to bolster economic security is emblematic of a broader globalization backlash, one that has increasingly received scholarly treatment [12–16]. Existing studies, however, offer little evidence for a connection between public opinion and globalization’s retreat. For instance, though anti-globalization candidates and policies have achieved increased success, reviews of public attitudes toward globalization (e.g., [17]) show little change in support for free trade and investment in the United States. While scholars have examined political attitude formation in the context of highly salient offshoring events [18, 19], they have been relatively silent on the topic of reshoring.

In this research, we investigate the extent to which the public supports or opposes reshoring, and the basis of such attitudes. We draw on the political economy, security, and public opinion literature to develop and test the factors that shape public opinion of reshoring.

We pre-registered hypotheses and analyses related to security and economic self-interest considerations and carried out a two-part survey experiment on diverse samples of Americans. In the first study, we varied the country from which manufacturing was to be reshored (China or South Korea) and the product to be reshored (steel or semiconductors). Our theory anticipated that support would be higher for reshoring from China (an adversary) and for semiconductors (a salient product with direct consumer impact in terms of price and availability). We selected steel as a traditional industry subject to longstanding political discourse about offshoring and reshoring—a reasonable control group compared to semiconductors, as we discuss subsequently. Section 5 in S1 Appendix contains a discussion of the information environment at the time of our studies. We found that support for reshoring among Americans was high in absolute terms regardless of the country from which goods were to be reshored, the product to be reshored, and respondents’ political party (Democratic or Republican). This suggests that underlying support for de-globalization policies is quite high despite significant elite and public polarization in American politics more generally, including on foreign policy issues [20, 21].

The results from the first study motivated the second, which sought to understand the basis of such high support for reshoring. We probed potential mechanisms of support, investigating whether respondents were motivated by a desire to bring jobs back to the US, consumer price concerns [22, 23], a feeling of nostalgia for past American superiority in manufacturing [24, 25], or xenophobic skepticism toward foreign out-groups. To weigh such mechanisms, we manipulated several additional factors: price increases (low or high), the types of jobs created (blue or white collar), and the investment partner (China or Canada). We found evidence that Americans are sensitive to large price increases, and they are less willing to reshore from nearby allies like Canada, whose qualities such as demographic composition impact respondents’ perceptions of “foreignness” [26, 27].

In sum, reshoring has become a bipartisan pillar of American economic policy in the last decade, accelerating in recent years. While the literature on reshoring has begun to catch up to such policy shifts [28, 29], scholarship has been relatively silent on its microfoundations. The sustainability of reshoring policies hinges not just on levels of support but shifts in the factors that shape it—whether cost, geography, or type of industry. Our analysis, which finds high support for reshoring in absolute terms regardless of the conditions assigned and across partisan divides, suggests that these policies have a strong public backing. However, we also find that such support is cost-sensitive: if price increases from reshoring intensify, it could soften approval of these policies.

### Hypothesizing public attitudes toward reshoring

Reshoring, as we note in the introduction, involves bringing manufacturing back to one’s home country. The concept overlaps with several strands of political economy scholarship, including work on globalization, trade, and investment. We contribute to each and discuss them in turn.

We begin with globalization, in which industries, consistent with expectations of comparative advantage, shift their production abroad to reduce manufacturing and labor costs. Reshoring then is a form of *deglobalization*, involving retrenchment from international trade and investment in favor of localized production. Reshoring is also linked to international trade because it aims to internalize the production of goods domestically rather than importing them from foreign suppliers. The same is true for foreign direct investment (FDI)—reshoring may reduce outward FDI by shifting production home from abroad, and it may create incentives (e.g., through subsidies) that seek to boost inward FDI.

We draw most heavily on the literature examining public opinion of trade. We do so because government policies designed to encourage reshoring—e.g., increased tariffs on imported goods and new subsidies for local manufacturers—are aimed at reshaping trade relations. When companies move production back home, they reduce reliance on imports of finished goods, often in pursuit of reduced trade deficits. If subsidies bolster the international competitiveness of domestic goods, increased production may also bolster exports. Further, the process of reshoring often involves a reevaluation of supply chains, where companies seek to minimize logistical costs and improve responsiveness to market demands. Such a focus on local sourcing can lead to a decrease in imports and promote the use of domestic suppliers, also significantly altering the flow of trade.

Of course, reshoring also shakes up investment relationships (e.g., by promoting inward FDI and reducing incentives to engage in outward investments). Our analytical framework thus also borrows insights from the FDI literature. Research on FDI increasingly considers the importance of threat perception and reciprocity in shaping attitudes towards FDI and investment restrictions [30–32]. In doing so, it builds on a large body of work on the public opinion of trade, which speaks more directly to the importance of geopolitical ties. We argue that such security considerations are are decisive in the current debate over semiconductor production. Indeed, reshoring production from an adversary implies the severing of a trade relationship as well as reduced outward investment. We also draw on the deglobalization literature, insofar as the framing of reshoring as a way to bolster economic security is emblematic of a broader globalization backlash.

In conceiving of reshoring as fundamentally reshaping trade relationships, we connect with a large body of work on the public opinion of trade. First, we theorize that security concerns weigh on public attitudes. Reshoring production from an adversary implies the severing of a trade relationship. We observe that the public will be more receptive to reshoring when the investment partner is an adversary than when the partner is an ally. Research recognizes that economic relationships, from aid flows to membership in international organizations, often mirror security relationships [33–35]. Such patterns are attributed to at least two considerations. Countries might use the economic gains from trade and investment relationships to bolster their militaries [33, 34]. These concerns resonate with the public, which has expressed preferences for trade and investment with allies over adversaries in part due to the association between trade gains and military advantages [31, 36].

Therefore, we expect a preference for reshoring from an adversary to deny that country the economic benefit. Given the growing skepticism of and competition with China in the US and the multi-decade economic and security partnership with South Korea, we select these two countries as representative adversary and ally for our survey, respectively. We specifically hypothesize:

**H1:** *Respondents will be more likely to support reshoring when the partner is China than when the partner is South Korea.*

Second, we posit an important role for economic self-interest concerns. Existing research notes, “the theoretical propositions originating in macroeconomic theories suggest that material self-interest should be dominant in preference formation” [37, 1266]. The argument goes that individuals are egocentric, and international economic policies have distributional consequences that are potentially directly relevant to one’s pocketbook or material well-being in general [38]. Literature in this vein broadly suggests that individuals will support an economic policy when they see economic benefit to doing so [22, 23, 39].

Though scholars have long shown that individuals have difficulties pinpointing the downstream economic consequences of trade policies [40, 41], recent work suggests that individuals may do so when the information environment, inclusive of media and elite cues, provides them the requisite information [42]. We build on this literature, highlighting the importance of information for individuals’ assessments of trade policy and reshoring in particular.

Although Americans experienced shortages of many types during the pandemic, the effect of semiconductor shortages was both novel in scope and uniquely impactful for consumers [43]. In July 2021, the Wall Street Journal reported that “the chip shortage is shaping up to be the dominant theme of yet another earnings season for the automotive industry” [44]. In September of that year, the Journal noted that Toyota would be idling a third of its factory capacity because it lacked a sufficient supply of chips [45]. Later that month, the Journal wrote that “the once-obscure world of automotive microchips will never be the same again” as manufacturers worked to avoid repeating the “mess” that had affected the availability of new cars and caused widespread price hikes [46]. S&P Global similarly showed that 5G smartphones had increased in price by 468 percent between 2019–2020, again as a result of chip shortages [47]. Semiconductor chip supply thus became top-of-mind for the average consumer as products like cars, gaming consoles, and home electronics were delayed or sold at higher prices due to limited chip supply. In late 2021, the Biden administration began calling for funding to secure the semiconductor supply chain [48].

To explore the effect of economic self-interest arguments, we contrast public attitudes toward reshoring two different industries, comparing support for reshoring semiconductor chips with steel production. Steel and semiconductors are necessary for the production of security-relevant items, including land vehicles and military aircraft. The manufacturing of both are similarly comparative disadvantaged industries in the United States. However, the chip shortages, as noted above, were both particularly salient and directly impacted consumers during the period under study because of their connection to cars, appliances, and smartphones that Americans use on a day-to-day basis. This is in contrast to steel, which was not subject to supply chain shocks during the period under study. As such, we specifically hypothesize the following:

**H2**: *Respondents will be more likely to support reshoring when the industry is semiconductors than when the industry is steel.*

### Initial test of attitudes toward reshoring

We administered a survey experiment to a diverse sample of 2,000 Americans in December 2021 using Lucid (for discussion of ethical principles, see Section 7 in S1 Appendix). We offer further discussion of respondent screening procedures in Section 2 in S1 Appendix. We also follow best practices for screening out low quality responses; we offer further explanation in Section 3 in S1 Appendix. Power analyses for both studies appear in Section 8 in S1 Appendix. Our final data set includes just over 1,500 responses.

We randomly assigned participants to one of four conditions. Respondents received information about one of two industries (steel or semiconductors) and one of two countries (China or South Korea). The specific text of the vignette was as follows: “In recent years, the United States has lost global market share of the [semiconductor or steel] manufacturing industry to [China or South Korea]. The United States government has periodically proposed incentives and investments to reshore more manufacturing—in other words bring more manufacturing home—from [China or South Korea].” While there are several ways to promote reshoring, including tax breaks, subsidies, tariffs, and import controls, we are agnostic about the specific government policy. We instead used more general language that mirrors media coverage and political statements, emphasizing government “incentives and investments” [49]. Semiconductors have been and remain salient in media and political discourse; modeling our vignettes on such language increases external validity and ensures our vignettes are similar to information salient in the public zeitgeist.

Our dependent variable is measured based on responses to the following question: “Do you support or oppose the series of incentives and investments to prop up the US [steel or semiconductor] industry?” Respondents answered on a five-point scale from “Strongly oppose” to “Strongly support.” We also asked standard demographic questions as well as a nationalism question, posed as follows: “When someone says something bad about the American people, how strongly do you feel it is as if they said something bad about you?” and it is measured on five-point scale from “Not strongly at all” to “Extremely strongly.” This measure captures national attachment; existing work convincingly shows national attachment is a leading driver of attitudes towards globalization and is thus appropriate here [50].

Descriptive statistics for our sample can be found in Table A1 in S1 Appendix. Fig A4 in S1 Appendix, meanwhile, shows how our dependent variable is distributed across respondents. Last, a detailed description of relevant survey questions can be found in Section 3 in S1 Appendix. This research was declared exempt by the IRB at Cornell University, protocol number 2111010734. All respondents were at least 18 years of age and gave their written consent before the survey began. The recruitment period for study 1 was December 5–7, 2021. The recruitment period for study 2 was August 31–September 7, 2022.

**Fig 1:**
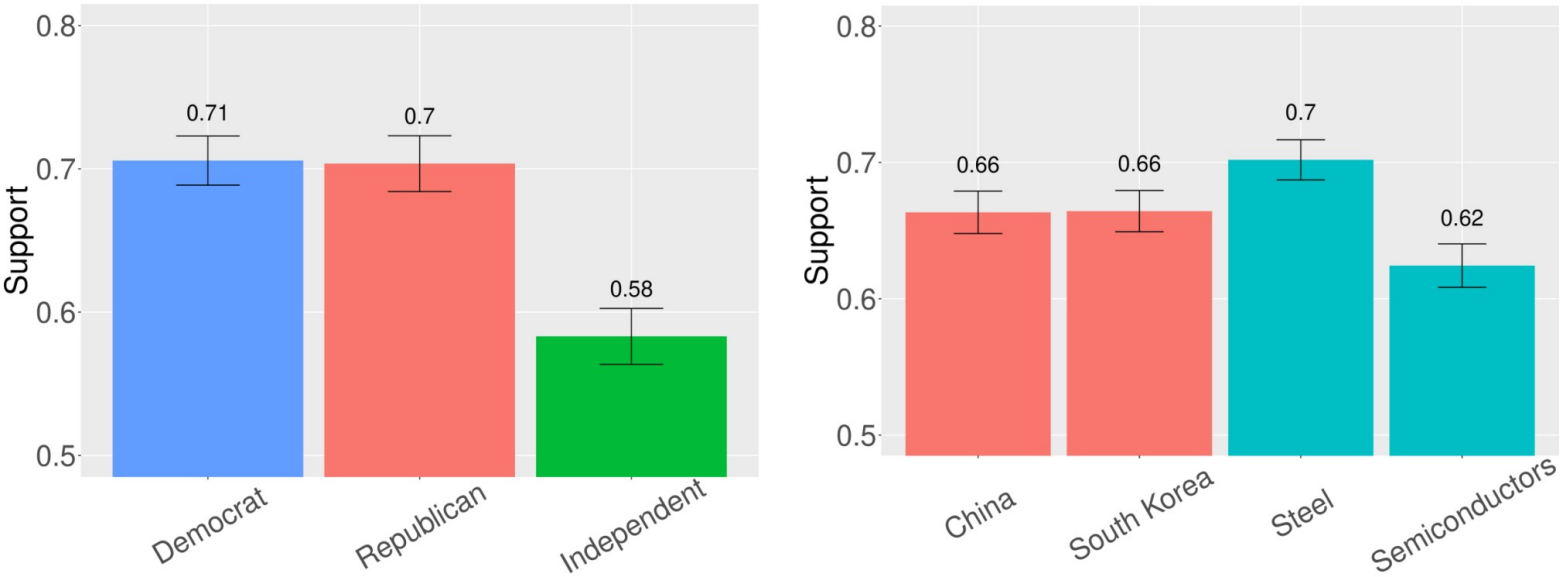
Support for reshoring by treatment and party (Experiment 1). Fig 1 shows the share of respondents that offered support for reshoring by responding either “Strongly support” or “Moderately support” to our outcome question. 95 percent confidence intervals are included. The sample includes 701 Democrats, 550 Republicans, and 644 Independents. (a) Treatment, (b) Partisanship.

To start, we examine levels of support broken out by treatment arm. These results are shown in panel (a) of Fig 1. Americans support reshoring from both China and South Korea—66 percent of respondents expressed support for bringing manufacturing home from each country despite apparent differences in security ties between them and the United States. Moreover, we find evidence that US citizens are more supportive of reshoring the production of steel than semiconductors, though support for reshoring both remains high in absolute terms. This findings perhaps suggest an important role for nostalgia politics [9, 51], or an expressed desire for the US to return to an era of past manufacturing prowess. We further probe this explanation in our follow-up study.

We also calculate the percent of respondents that support reshoring across party affiliations. Strikingly, we do not identify major differences based on respondents’ partisanship; average support for reshoring is nearly identical among Democrats and Republicans, as panel (b) of Fig 1 shows, although considerably lower for Independents. These patterns mirror bipartisan elite consensus on reshoring chips. In an era of heightened polarization [52, 53], reshoring appears to be a rare area of partisan agreement. This is surprising, in part, because Republicans tend to be concerned about government spending initiatives [54, 55].

Next, we more systematically test the main effects of our treatments on respondents’ levels of support for reshoring. With respect to H1, we find a treatment effect of 0.042 (p = 0.163) for the China treatment relative to the South Korea condition. With respect to H1, we do not detect an effect of the China treatment relative to the South Korea condition (0.042, p = 0.163). Throughout, we report bootstrapped treatment effects from 1,500 draws, though results are nearly identical in each case for a simple two-tailed difference-in-means. With respect to H2, we find a *negative* and statistically significant treatment effect for semiconductors versus steel (-0.115, p = 0.002). This corresponds to a roughly three percent decrease in support for reshoring when moving from the steel to semiconductor condition. We also model our results in a regression framework, both with and without the inclusion of relevant covariates—see Table A3 in S1 Appendix.

### Follow-up study

Motivated by the results of the first survey, we derived three additional pre-registered hypotheses to better understand why Americans are supportive of reshoring manufacturing and what factors moderate that support.

First, we explored why support for reshoring was high both when the target country was an ally (South Korea) and adversary (China). Scholars suggest that individual trade preferences are sociotropic: individuals base their support for trade on the potential consequences for the community rather than oneself [39]. They argue that ethnocentrism can play a role and individuals may have “a tendency to think less of those who are racially or ethnically different from one’s own group” [39, 438]. Applying those insights to our research question, we hypothesize that one reason respondents were equally supportive of reshoring from South Korea and China is that both are in Asia and could be viewed as a racial or ethnic “other” to the median American.

If this logic is correct, Americans might be less eager to reshore production from a country that is more demographically similar to theirs than either country in Asia. Canada, for example, is a demographically similar and geographically proximate ally of the United States. Shared borders and similar demographic makeups should reduce feelings of “foreignness” relative to countries in Asia [27]. It is also possible that respondents anticipate positive economic spillovers from production in Canada as a result of geographic proximity to the US, though we believe this to be unlikely. Individuals typically struggle to ascertain whether and when trade is beneficial versus harmful for economic well-being in general [41], let alone whether production in one country might create economic opportunities in a bordering country.

In the second survey, we therefore selected country comparisons that allowed us to contrast our findings with the results from the first survey to observe whether racial, ethnic, and geographic differences alter reshoring perceptions, holding constant alliance status. While there are other factors that vary between Canada and China, including factor endowments and levels of development, respondents would need to possess especially sophisticated preferences about trade to recognize these differences [40, 41], and we believe this to be unlikely. Taken together, we expect the following:

**H3:** *Respondents will be more likely to support reshoring when the trade partner is China than when the trade partner is Canada.*

Second, Americans might be concerned about economic self-interest but in ways that we could not easily capture in the first survey. Reshoring drives price increases over the medium-term because manufacturing is moved away from comparative advantage countries where labor and inputs are typically cheaper. If economic self-interest drives attitudes, Americans may be concerned about such price increases, especially given high inflation that emerged during the period under study. Notably, price increases stemming from reshoring can be severe [28, 56]. Such economic concern could manifest at the individual-level or in skepticism about the state of the national economy as a whole [39]; both are compatible with an economic self-interest story. We therefore introduce information about price, with respondents receiving information about price increases of either 10 or 25 percent as a result of reshoring, and we expect Americans to be sensitive to, and less supportive of, reshoring as prices increase.

**H4:** *Respondents will be less likely to support reshoring when it is associated with price increases, with support declining in the magnitude of the price increase.*

Third, we consider sociocultural factors. In particular, we examine whether support for reshoring is associated with nostalgic sentiment—a yearning for a period of past American manufacturing dominance [9]. Scholars have suggested that resurgent populism in the West is driven, in part, by a “cultural backlash” against globalization [17]—an embrace of a nation’s history, which often favors one racial or ethnic category [57].

In our case, if sociocultural factors affect perceptions of reshoring, respondents should wish to protect and grow jobs that are perceived to be particularly “American,” which scholars map onto blue-collar industries that involve working with one’s hands [25]. We capture this by manipulating whether respondents receive information about the effect of reshoring on blue or white collar jobs. Support for the creation of blue over white collar jobs, holding job creation itself constant, would be indicative of a desire to return to an era of past economic greatness in manufacturing rather than to leverage capital abundance and comparative advantages [22, 23]. Some scholars have directly studied the role of respondent job type and subsequent support for capital inflows [58]. We probe this link further in the sections following the initial analyses: the heterogeneous treatment effects discussed therein suggest that respondent job type modulates the effects of our treatment conditions.

**H5:** *Respondents will be more likely to support reshoring it is associated with an increase in blue-collar jobs rather than white-collar jobs.*

The dependent variable in our analysis is the same as the first study; full question text can be found in Section 3 in S1 Appendix. Descriptive statistics appear in Table A4 in S1 Appendix, and bar graphs of support for reshoring can be found in Fig A4 in S1 Appendix.

**Fig 2:**
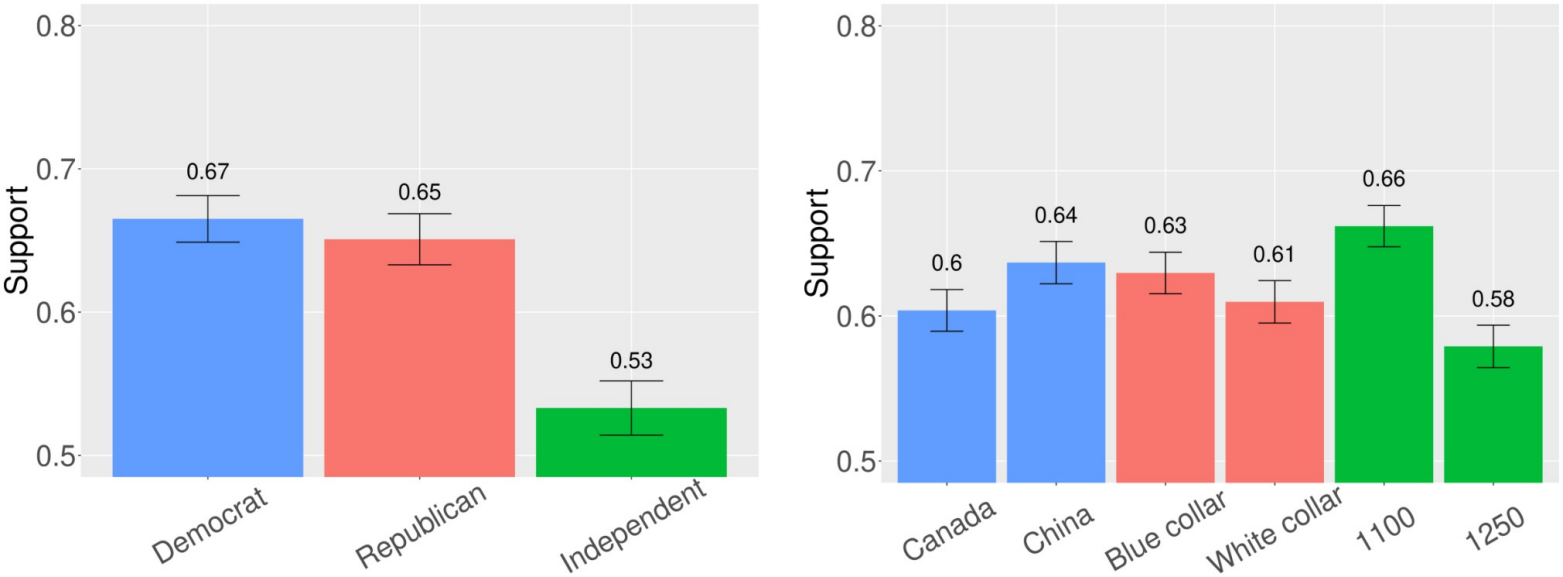
Support for reshoring by treatment and party (Experiment 2). Fig 2 shows the share of respondents that offered support for reshoring by responding either “Strongly support” or “Moderately support” to our outcome question. Panel a shows treatment effects, including country; white versus blue collar; and price increase based on a standard iPhone cost with 1,100 versus 1,250 dollars corresponding to the 10 and 25 percent increases. Panel b shows heterogeneous effects by party. 95 percent confidence intervals are included. The sample includes 834 Democrats, 721 Republicans, and 699 Independents. (a) Treatment, (b) Partisanship.

To begin, we present the results by treatment arm (Fig 2, panel a) which reveals variation that largely aligns with our expectations. Support for reshoring is higher when the source country is China (64 percent) than Canada (60 percent). The treatment effect on the five-point scale associated with our dependent variable is 0.104, and this difference is statistically significant (p = 0.008). This finding suggests Americans judge allies differently depending on their geography and demographic makeup—there may be support for reshoring from distant allies that are perceived to be more “foreign,” but such support wanes some for proximate and demographically similar allies.

Next, 63 percent of respondents support reshoring when it generates blue collar jobs, while only 61 percent support it when it creates white collar jobs; the difference on our five-point scale fails to achieve statistical significance at conventional levels, though it points in the expected direction (effect = 0.046, p = 0.144).

The largest effect, however, is driven by the price increase associated with reshoring—only 58 percent support bringing manufacturing home when it results in a 25 percent price increase, though 66 percent are supportive when the price increase is only 10 percent. The effect size here is 0.159 on a five-point scale (p = 0.0007). Substantively, the China effect corresponds to a 3 percent increase in support for reshoring, and the price effect corresponds to a 4.5 percent increase. Though this was not pre-registered, we also looked ex post for interaction effects between our country and price treatments and detected none (effect = -0.007, p = 0.929 for interaction between China and 25 percent price increase treatments). Since respondents are not significantly more supportive of reshoring to create blue versus white collar jobs, and given their sensitivity to price increases, it appears respondents are more attuned to economic self-interest than nostalgia politics in the context of semiconductors. This is despite the fact that nostalgia may have driven support for reshoring steel in the first study.

We also disaggregate the results by partisanship, as is illustrated in panel b of Fig 2. Democrats and Republicans once again express similar levels of support for reshoring, and Independents remain more skeptical of moving semiconductor manufacturing to the United States. Still, reshoring appears to be a broad area of bipartisan consensus in an otherwise polarized polity. We similarly present our results in a regression framework, both with and without the inclusion of relevant covariates, and the findings are unchanged (Table A4 in S1 Appendix).

**Fig 3:**
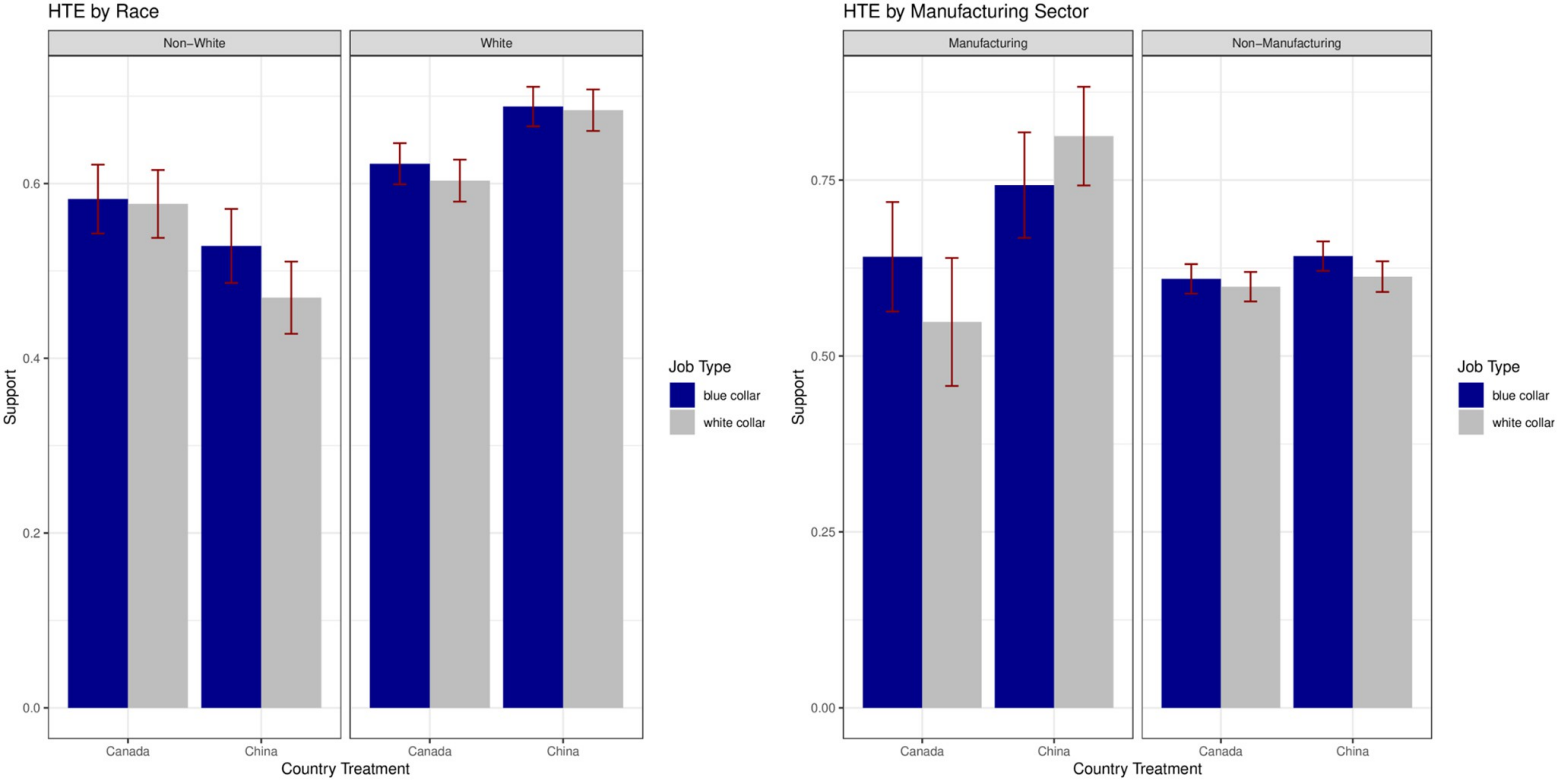
Heterogeneous treatment effects by race and manufacturing sector. Fig 3 shows mean standard errors of the country and jobs treatments across different subgroups. (a) HTE by Race, (b) HTE by Manufacturing Sector.

Finally, we conduct ex-post analysis of heterogeneous treatment effects to further tease out mechanisms; this analysis was not pre-registered but helps us to further assess the determinants of support for reshoring. We find that support is highest among two sub-groups—those employed in manufacturing and white respondents—as illustrated by Fig 3. In the eyes of those in the manufacturing sector, China may be perceived as the main country to which manufacturing jobs fled in recent decades. The impact of Chinese imports on areas that served as former US manufacturing hubs has been severely negative [12, 18], driving economic volatility and political polarization [13]. It is no surprise then that these individuals are especially eager to reshore semiconductor manufacturing from China. Notably, manufacturing workers are also enthusiastic about reshoring blue collar jobs from Canada, likely because they work in blue collar sectors themselves. These results indicate sensitivity to economic self-interest considerations among manufacturing workers and connect with work on how one’s industry of employment affects their attitudes towards foreign investment [58].

We also find that support for reshoring is much higher among white respondents than non-white respondents. Declining industries subject to job losses amidst globalization disproportionately employed white men at their peak [4]. White respondents may identify reshoring as a means to bolster the economic welfare and social status of their racial in-group. The race results also point to an important role for perceptions of “foreignness” at the individual-level. Non-white respondents are more supportive of reshoring from Canada—a predominantly white country with similar demographic make-up to the US—compared to China while the opposite is true for white respondents. This offers strong support for our posited ethnoracial mechanism and helps explain why support for reshoring from China is higher than support for reshoring from Canada in the aggregate when no such gap was present in Study 1 between China and South Korea.

## Discussion

Reshoring, which seeks to bring manufacturing back to domestic shores, accelerated in recent years. Though scholars have begun to take stock of this trend [28, 29, 59], the microfoundations of reshoring—what Americans think about the policy shift and which levers might moderate such attitudes—have not received sustained scrutiny. We address this gap in the literature and unpack how Americans assess reshoring, including why and under what conditions they are supportive of such deglobalization policies. This is important since policy shifts are more sustainable in democratic settings when they have requisite public backing.

Our findings suggest that Americans broadly support reshoring and that levels of such support depend little on partisanship, the affected industry, the country from which jobs are reshored, and whether blue- or white-collar jobs are created. This evidence starkly contrasts conventional wisdom, which typically identifies a partisan element to the globalization backlash, and especially right-wing responsiveness to trade shocks [12]. However, we identify some moderation in cases where reshoring provokes large price increases or production is moved from a demographically and geographically proximate ally. We further uncover heterogeneous effects contingent on respondents’ race and industry of employment, consistent with recent work [4].

Beyond the paper’s academic contribution, the research also has important policy implications, especially as the United States ramps up reshoring and escalates export restrictions on China. First, our results suggest that “friendshoring”—relocating manufacturing processes to countries that are considered geopolitical allies or “friendly” nations—may help the US balance the economic benefits of offshoring with the public’s preference for economic integration with geographically and demographically aligned allies. Second, our findings imply that reshoring may face headwinds if consumer prices tick up, given the cost sensitivity of the American public, especially as a result of comparatively high labor costs. Indeed, firms moving production back to the US have struggled with labor shortages that ultimately would raise costs, the reason why these industries originally reshored in search of cheaper labor [60].

While our study takes important steps toward understanding the basis of public attitudes toward reshoring, it does have some limitations that create fruitful opportunities for future research. For example, while we focus on semiconductors, policymakers have also moved to reshore aspects of other industries such as pharmaceuticals, clean energy, and electric vehicles. Whether our findings extend to other industries is an empirical question that is relevant to ongoing policy debates about supply chain resilience, re-igniting manufacturing sectors hollowed out by globalization, and inflationary pressures in the economy.

Lastly, while we made the conceptual claim that reshoring maps most closely onto dynamics associated with trade relationships and withdrawal of trade through policies such as protectionism, we urge more theoretical study along these lines. We acknowledge some conceptual fuzziness that conflates changes in trade and investment policy—specifically the notion that reshoring involves both trade and investment restrictions. Future work could try to tease out these dynamics more fully, for example exploring whether an American firm building a plant in the United States elicits more support than a Taiwanese-owned firm taking advantage of American incentives to build a plant in the United States (with a combination of American and Taiwanese labor). As policies intended to reshore manufacturing in critical goods evolve, so should the theoretical and empirical exploration of these questions.

## Supporting information

S1 Appendix Supplementary analyses. (PDF)
