# Supplementary material for: The political economy of reshoring: Evidence from the semiconductor industry
Source: PLoS One. 2025 Feb 14;20(2):e0316473. doi: 10.1371/journal.pone.0316473 (PMC11828386; doi:10.1371/journal.pone.0316473)
Supplement: S1 Appendix — (PDF) [file pone.0316473.s001.pdf]

The Political Economy of Reshoring: Evidence from the Semiconductor Industry

S1 Appendix

Contents

1 Pre-Registration **2**

2 Lucid, COVID-19, and Respondent Screening **2**

3 Survey Procedures and Questions **2**

3.1 Vignette (Survey 1) 2

3.2 Vignette (Survey 2) 3

3.3 Dependent variable (Surveys 1 and 2) 3

3.4 Covariates 3

4 Qualtrics **4**

5 Information Environment **4**

6 Supporting Statistical Information **6**

7 Discussion of Ethical and Human Subjects Principles **8**

8 Power Analysis **11**

1 Pre-Registration

All hypotheses were pre-registered with AsPredicted. An anonymous PDF of the pre-registration for the first two hypotheses can be found here: https://aspredicted.org/1Z8_4WY. An anonymous version of the pre-registration for the last three hypotheses can be found here: https://aspredicted.org/K81_TKC.

2 Lucid, COVID-19, and Respondent Screening

Our first study was fielded using Lucid's "Theorem" respondent pool. Lucid uses a quota system to target a nationally representative sample. Survey samples recruited with Lucid track well with US national benchmarks and are increasingly used in social science research, not for descriptive work where probability samples are needed, but for assessing relationships between variables, as is the objective here [61; 62].

Given that our first study was fielded during the Covid-19 pandemic, we recognize that the pandemic could affect our results. Research studying the effects of the pandemic on survey quality and generalizability shows that respondent attentiveness declined during the pandemic among online sample populations, and researchers are advised to use attention checks to screen for quality respondents [63]. We use such pre-treatment checks in our study. That said, [64] find that studies conducted during the pandemic consistently replicate pre-pandemic studies, though for some the effect sizes are smaller, which suggests that the timing of our study may yield more conservative estimates than if we had conducted the same study before the pandemic.

All respondents were provided with an informed consent form and had the option to opt-out of the study. Consistent with best practices for data quality [65], respondents were blocked from participating if they were located outside of the US or were flagged for using a Virtual Private Server (VPS). We collected responses until we had 2,000 respondents, as outlined in out pre-registration. We dropped respondents that wrote nonsensical free responses as an attention check (i.e., those that responded with one word or provided gibberish responses).

3 Survey Procedures and Questions

Following the practices outlined by [65], we implemented techniques to block respondents from participating if they could not verify they were located in the US or they were using a Virtual Private Server (VPS) to mask their location. We also had respondents answer a free response question unrelated to our study, which we used as an attention check. Respondents who wrote gibberish or who entered text that was unrelated to the question were removed from the sample, which resulted in 23 percent of the sample being excluded.

3.1 Vignette (Survey 1)

In recent years, the United States has lost global market share of the [semiconductor or steel] manufacturing industry to [China or South Korea]. The United States government has periodically proposed incentives and investments to reshore more manufacturing – in other words bring more manufacturing home – from [China or South Korea].

3.2 Vignette (Survey 2)

As you know, Americans buy products that are either made in foreign countries or have parts that are made in foreign countries. For example, the US currently imports semiconductors in large volumes from [China or Canada].

In recent months, these supply chains have become strained, and there is debate about whether the US should provide incentives and investments to American companies to bring their manufacturing home.

If implemented, supporters say these investments would bring large numbers of [blue or white collar jobs] back to the United States. But some skeptics of these proposals have suggested that they will increase prices of relevant goods by [10 or 25%] . This means that a smartphone, which currently costs $1000, would cost[$1100 or $1250].

As a summary:
- The US imports semiconductors from [Canada or China].
- Reshoring this industry would create [blue or white collar] American jobs.
- Reshoring this industry would drive [10 or 25%] higher prices for American consumers.

3.3 Dependent variable (Surveys 1 and 2)

- Do you support or oppose the series of incentives and investments to prop up the US [steel or semiconductor] industry? [Strongly oppose, Oppose, Neither support nor oppose, Support, Strongly support]

3.4 Covariates

We randomized the order of all covariate questions with the exception of the Trump voter variable, which we asked at the end of the survey to avoid differential attrition. Questions were the same for both surveys that we fielded unless otherwise noted.

- Nationalism: When someone says something bad about the American people, how strongly do you feel it is as if they said something bad about you? Measured on five-point scale from "Not strongly at all" to "Extremely strongly."
- Male: binary equal to 1 if respondent best identified themselves as male.
- Age (Survey 1): self-identified age in integer form.
- Age (Survey 2): self-identified age on a three-point scale (18-34, 35-54, 55+)
- Income: measured on 12-point scale from "$10,000 or less" to "$150,000 or more."
- Education: measured on eight-point scale from "Less than High School" to "Professional Degree (JD, MD)."
- Republican: binary equal to 1 if respondent self-identified as a Republican as opposed to a Democrat or Independent.

- Trump: the question asks “whom did you vote for in the 2020 presidential election?” Measured as binary equal to 1 if responded Donald Trump. We ask this question post-treatment to avoid concerns about differential attrition pre-treatment. We do not observe attrition anyway based on this question.

4 Qualtrics

Qualtrics delivered 2,254 quality completes. They removed poor-quality responses and ensured that each respondent was a US citizen and passed a basic attention check at the start of the study. The sample is representative of the US population by Census benchmarks for age, gender, and region. They did not collect partial responses. The median time to completion was 6 minutes. They also implemented a speeding check – measured as one-half the median soft launch time – which terminated those who were not responding thoughtfully.

5 Information Environment

Gallup found that a majority of Americans experienced supply chain problems in 2021, including shipping delays and price increases [68]. The industries for which such supply chain kinks were particularly acute should therefore be top-of-mind for citizens. The media, meanwhile, covered the semiconductor supply problem and connected it to consumer price inflation and low product availability; Figure A1 shows that semiconductors rose from relative obscurity to a more mainstream issue around the time of our studies, nearly converging with coverage on steel. Steel coverage, conversely, decreased from its historic high during the Trump Era. A similar plot with Google Trends data appears in Figure A2, with accompanying discussion. The result, then, was a high information environment in which consumers could ably connect trade policy over semiconductors to their own economic self-interest.

We compare support for reshoring semiconductor chips with steel production to help isolate the effect of short-term economic self-interest considerations. Steel and semiconductors are necessary for the production of security-relevant items, including land vehicles and military aircraft. The manufacturing of both are similarly comparative disadvantaged industries in the United States. We contend that Americans should put a premium on reshoring chip production because of the salient connection to the availability and cost of household goods such as cars, appliances, and smart phones. Steel also faced pandemic shortages but was less inextricably linked to the type of direct, consumer impacts that we expect individuals would associate with chip shortages. Though President Trump did seek to revive American steel production in 2018 by levying tariffs on Chinese steel and aluminum and instituting tax cuts for American producers [69]. Indeed, the media trends plot above suggests that news articles on steel and semiconductors nearly converge during the time of our studies.

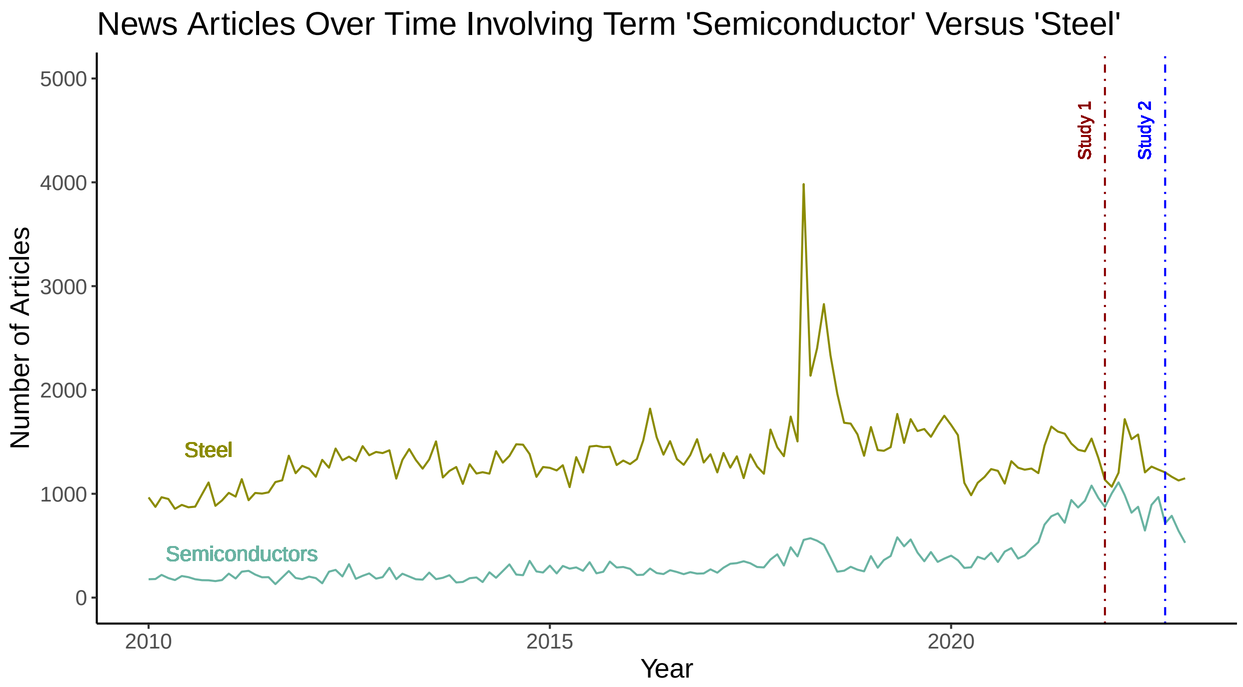

Fig A1: **Salience of Semiconductors versus Steel.** This figure shows public interest from AB/INFORM searches for news sources with the term "semiconductor" versus "steel" from the years 2010 to 2022. ABI/INFORM maintains a database of millions of articles across nearly 10,000 news outlets (especially business-oriented outlets, e.g., Wall Street Journal, The Economist). Vertical lines demarcate the timing of our two studies. Source: ABI/INFORM, https://about.proquest.com/en/products-services/abi_inform_complete/. Accessed 23 Apr. 2023.

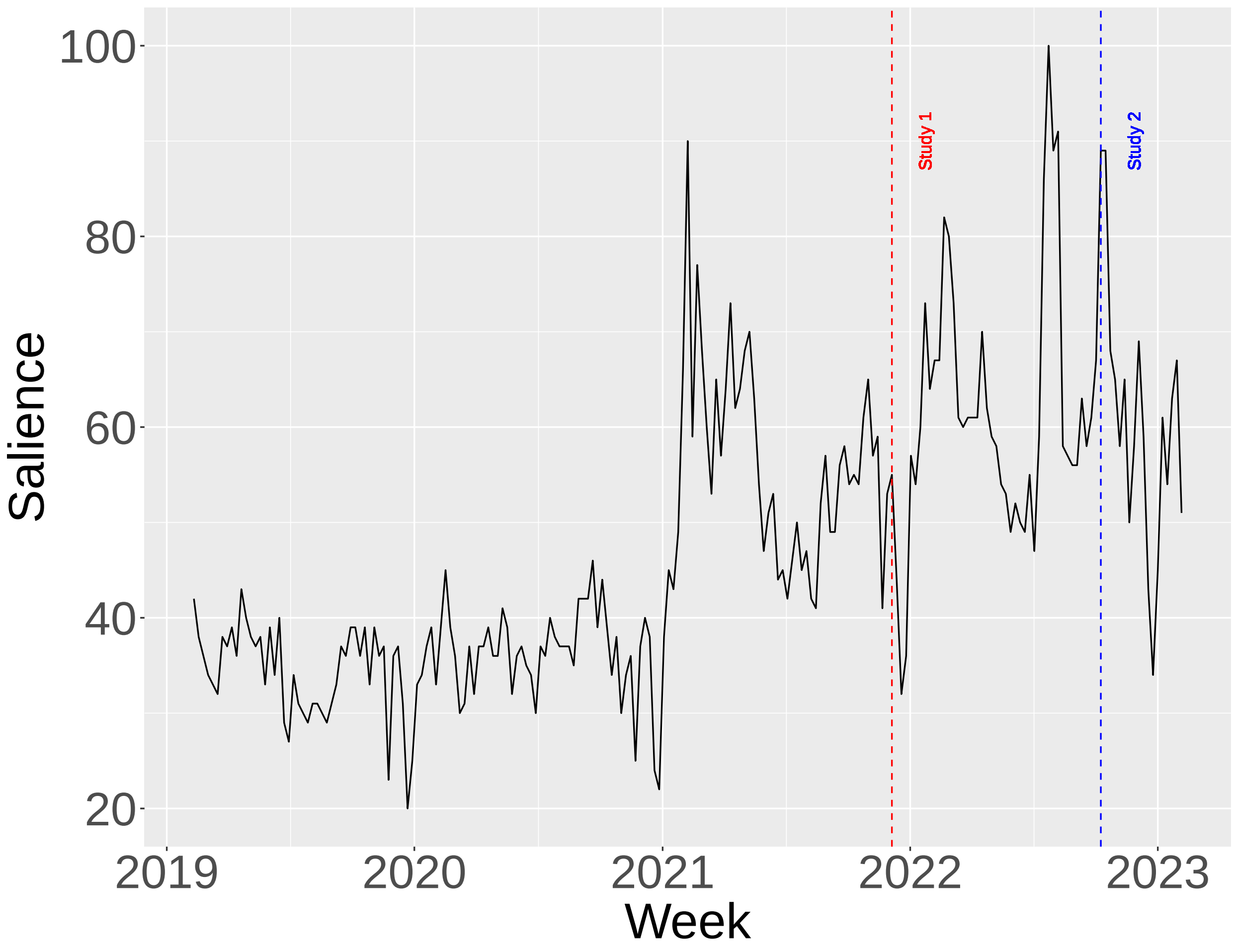

Fig A2: **Salience of Semiconductors (Google Trends).** This figure shows public interest from Google searches in semiconductors, which as a topic includes related search terms like chips. Data comes from Google trends. Vertical lines demarcate the timing of our two studies.

The Google trends plot in Figure A2 shows that semiconductors elicited public interest at the time of our studies. Notably, scholars have identified a link between Google trends and Gallup “most important problem" questions [66]. This suggests that Google trends are a good measure of issue salience. interest in and concern with chips increased in early 2021 when downstream industries, which produce goods for consumption such as laptops, cars, and household appliances, suffered from production shortfalls and delivery lags as a result of chip shortages. These industries, in turn, largely passed price increases resulting from such shortages onto consumers.

6 Supporting Statistical Information

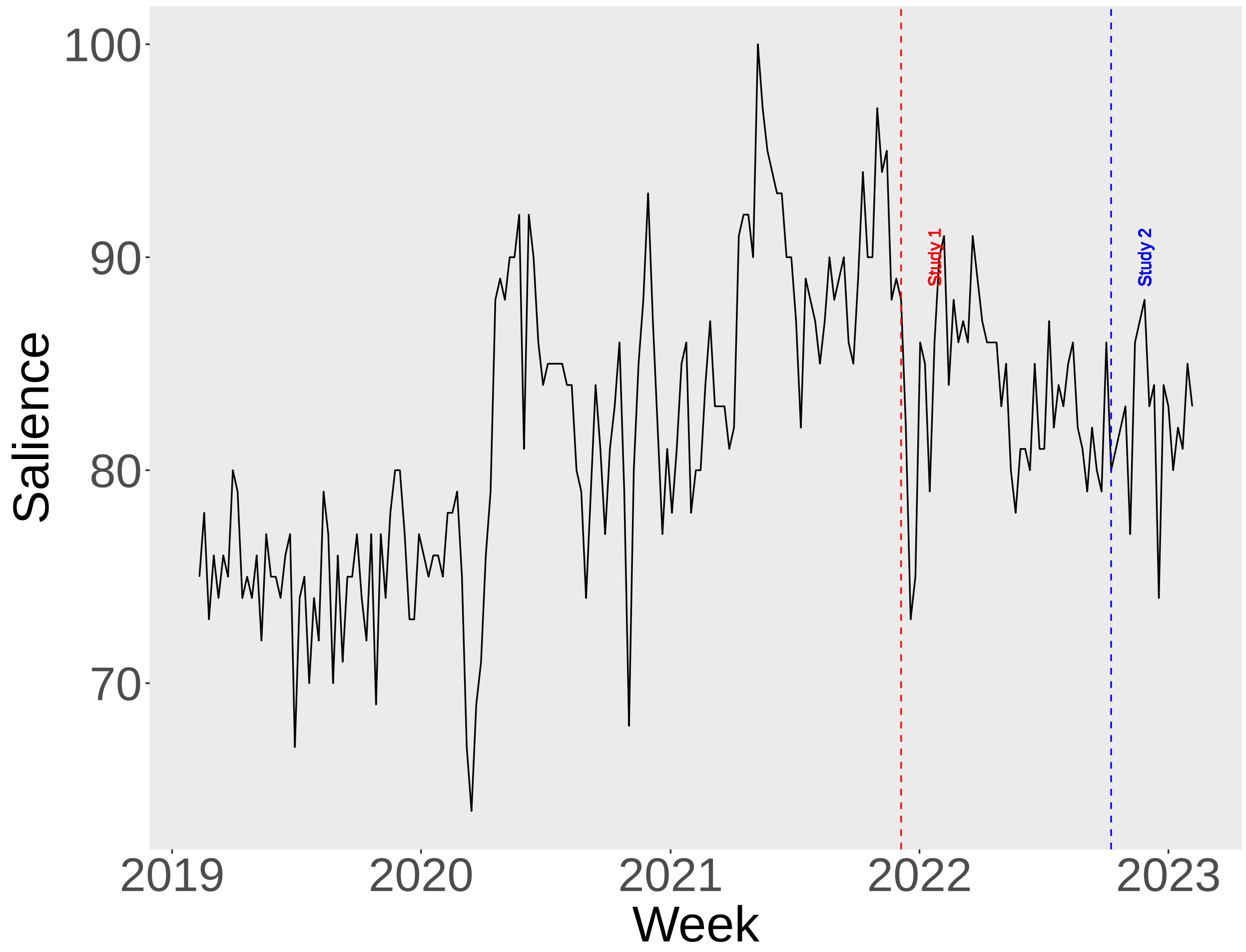

Fig A3: **Salience of Steel.** This figure shows public interest from Google searches in steel, which as a topic includes related search terms. Data comes from Google trends.

Statistic	N	Mean	St. Dev.	Min	Max
Reshoring	1895	3.89	0.91	1	5
Male	1895	0.48	0.50	0	1
Age	1895	46.28	16.82	18	94
Income	1895	5.91	3.36	1	12
Education	1895	3.72	1.54	1	8
Trump voter	1895	0.34	0.47	0	1
Republican	1895	0.29	0.45	0	1
Democrat	1895	0.37	0.48	0	1
Independent	1895	0.34	0.47	0	1

Table A1: **Descriptive Statistics (Survey 1).**

Statistic	N	Mean	St. Dev.	Min	Max
Reshoring	2254	3.74	0.98	1	5
Male	2254	0.45	0.50	0	1
Age	2240	2.10	0.81	1	3
Income	2254	5.54	3.28	1	12
Education	2254	3.59	1.53	1	8
Trump voter	2254	0.35	0.48	0	1
Republican	2254	0.32	0.47	0	1
Democrat	2254	0.37	0.48	0	1
Independent	2254	0.31	0.46	0	1

Table A2: **Descriptive Statistics (Survey 2).** Note that age is measured on a 1-5 scale corresponding to age ranges.

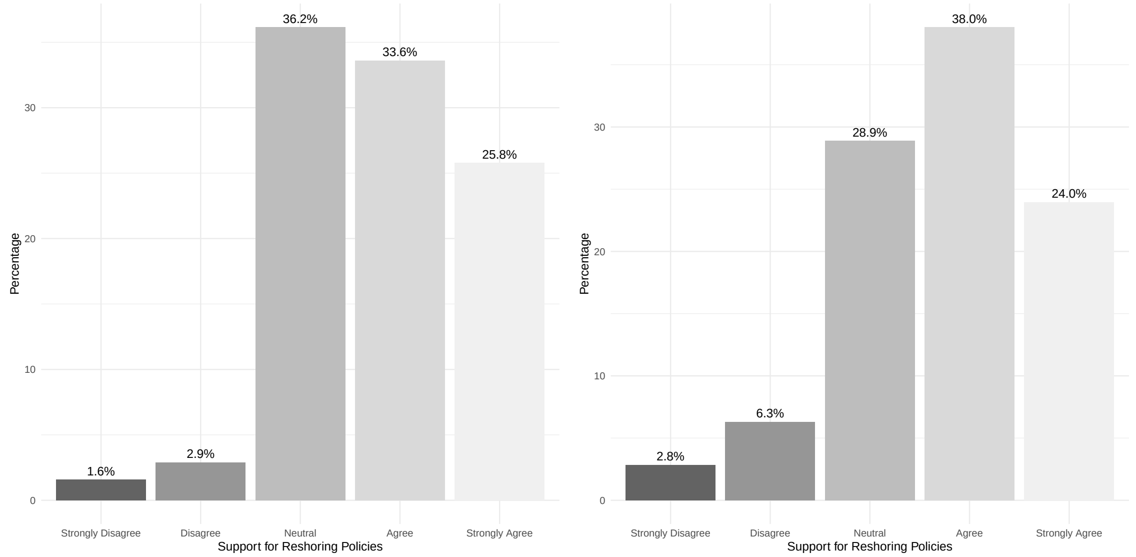

Fig A4: **Distributions of Dependent Variables.** Left: Survey 1. Right: Survey 2.

7 Discussion of Ethical and Human Subjects Principles

The human subjects research included in this paper complies the with Principles and Guidance for Human Subjects Research outlined by the APSA and was evaluated by the Institutional Review Board at the appropriate universities. The study was fielded with the survey firm Lucid and was reviewed by the IRB at [Name Redacted] (Protocol #: [Number Redacted]) and was declared exempt. The risks to subjects were evaluated to be minimal and the researchers took steps to ensure that any potentially identifying information was protected and then redacted prior to making the data available for analysis and replication. There were no conflicts of interest identified for the researchers. The data for replication will be made available when the manuscript is published.

	Support for reshoring	
	(1)	(2)
China	0.045	0.027
	(0.042)	(0.041)
Semiconductor	-0.116^{***}	-0.099^{**}
	(0.042)	(0.041)
National superiority		0.087^{***}
		(0.033)
Male		0.154^{***}
		(0.042)
Age		0.005^{***}
		(0.001)
Income		0.013^{*}
		(0.007)
Education		0.044^{***}
		(0.015)
Republican		-0.001
		(0.057)
Trump voter		0.178^{***}
		(0.056)
Constant	3.925^{***}	3.097^{***}
	(0.035)	(0.107)
Observations	1,895	1,895
Adjusted R^2	0.004	0.055
Note:	$^{*}p<0.1$; $^{**}p<0.05$; $^{***}p<0.01$	

Table A3: **Experiment 1 Results.**

For these studies, respondents were initially asked to complete an electronic standard adult consent form that informed them they were being asked to participate in a voluntary study that had been evaluated by [Redacted University Name] institutional review board. The consent form informed respondents they would be asked a variety of questions about their background, political preferences, and thoughts on government policies, the estimated length of time, the contact information for the investigator, and that the study was deemed to be of minimal risk. Respondents could select "If you wish to participate, please click the 'I Agree' button and you will be taken to the survey." or "If you do not wish to participate in this study, please select 'I Disagree'." If the latter was selected, the survey was terminated.

As noted above, respondents were recruited by the survey company Lucid. Respondents vol-

	Support for reshoring	
	(1)	(2)
China	0.098**	0.097**
	(0.041)	(0.041)
Blue collar	0.046	0.043
	(0.041)	(0.041)
High price	−0.154***	−0.148***
	(0.041)	(0.041)
National superiority		0.079**
		(0.032)
Male		0.191***
		(0.044)
Age		0.130***
		(0.027)
Income		0.024***
		(0.007)
Education		0.041***
		(0.015)
Republican		0.017
		(0.057)
Trump voter		0.024
		(0.056)
Constant	3.747***	2.886***
	(0.042)	(0.113)
Observations	2,254	2,240
Adjusted R^2	0.008	0.043
Note:	*p<0.1; **p<0.05; ***p<0.01	

Table A4: **Experiment 2 Results.**

untarily choose to opt in to participate in the survey firm's panel and are compensated based on the terms of the survey vendor, which can include cash, gift cards, and loyalty reward points. The respondents were all in the United States and respondents had free choice to opt in or out of the survey firms' panels. Respondents also had the opportunity to contact the researcher regarding any concerns about compensation or the survey itself, but we did not receive any complaints or concerns from participants. Our study does not engage in deception since each component of the experiment provided factual information about the policies in question.

With regard to Principal 10 on the impact of the research on the political processes, we do not believe there is any reason to believe that our studies would have had an impact on political processes such as elections or policy creation. Respondents were only asked their opinion on the subject of reshoring. We therefore do not see the survey as presenting any information to respondents that would alter their political behavior or political processes.

8 Power Analysis

We present the results of a post hoc power analysis for our two survey waves using the figures presented in Table A3 and Table A4. For both waves, we use an $\alpha = 0.05$ for the probability of Type I error. Because we are using a post hoc power analysis, we use F tests from multiple regression (R^2 deviation from zero); however, we constrain the power analysis to the models without non-experimental covariates. We calculate these figures using the open-sourced "G-Power" statistical software package [67].

For the first study wave in December of 2021, our power was $1 - \beta = 0.694$. For our second survey wave disbursed in September of 2022, our power was $1 - \beta = 0.960$. These results suggest that, while the second study wave was highly powered, the first survey was only moderately powered.

References

[1] Barbieri P, Ciabuschi F, Fratocchi L, Vignoli M. What Do We Know About Manufacturing Reshoring? Journal of Global Operations and Strategic Sourcing. 2018;11(1):79-122.

[2] Reshoring Initiative. 2021 Data Report: Essential Product Industries Drive Job Announcements to Record High; 2021. Reshoring Initiative. Available from: https://www.reshorenow.org.

[3] Rodrik D. Populism and the Economics of Globalization. Journal of International Business. 2018;1:12-23.

[4] Baccini L, Weymouth S. Gone For Good: Deindustrialization, White Voter Backlash, and U.S. Presidential Voting. American Political Science Review. 2021;115:550-67.

[5] Consultancy Asia. Global Chip and Semiconductor Industry Heavily Reliant on Asia; 2021. Accessed: 21-Oct-2024. Available from: https://www.consultancy.asia/news/4074/global-chip-and-semiconductor-industry-heavily-reliant-on-asia.

[6] Semiconductor Digest. U.S. Aims for 20% of World,Äôs Leading-Edge Semiconductor Production by 2030; 2021. Accessed: 21-Oct-

2024. Available from: https://www.semiconductor-digest.com/u-s-aims-for-20-of-worlds-leading-edge-semiconductor-production-by-2030

[7] The White House. The Biden-Harris Plan to Revitalize American Manufacturing and Secure Critical Supply Chains in 2022; 2022. Accessed: 21-Oct-2024. Available from: https://www.whitehouse.gov/briefing-room/statements-releases/2022/02/24/the-biden-harris-plan-to-revitalize-american-manufacturing-and-secure-c

[8] Forbes Technology Council. Three Key Strategies to Drive American Manufacturing Reshoring; 2023. Accessed: 21-Oct-2024. Available from: https://www.forbes.com/councils/forbestechcouncil/2023/04/20/three-key-strategies-to-drive-american-manufacturing-reshoring/.

[9] Clark R, Khoban R, Zucker N. Breadwinner Backlash: The Gendered Political Consequences of Industrial Decline. IPES 2022 Annual Meeting. 2022.

[10] Reshoring Initiative. Reshoring Initiative February 2024 Newsletter; 2024. Accessed: 21-Oct-2024. Available from: https://reshorenow.org/content/newsletter/reshore-newsletter-202402.html.

[11] Schewe E. Semiconductor Shortages End an Era of Globalization. JSTOR Daily. 2021.

[12] Colantone I, Stanig P. The Trade Origins of Economic Nationalism: Import Competition and Voting Behavior in Western Europe. American Journal of Political Science. 2018;62(4):936-53.

[13] Autor D, Dorn D, Hanson G, Majlesi K. Importing Political Polarization? The Electoral Consequences of Rising Trade Exposure. American Economic Review. 2020;110(10):3139-83. NBER Working Paper 22637.

[14] Mansfield ED, Milner HV, Rudra N. The Globalization Backlash: Exploring New Perspectives. Comparative Political Studies. 2021;54(13):2267-85. Doi: 10.1177/00104140211024286. Available from: https://doi.org/10.1177/00104140211024286.

[15] Broz JL, Frieden J, Weymouth S. Populism in Place: The Economic Geography of the Globalization Backlash. International Organization. 2021;75(2):464-94.

[16] Ballard-Rosa C, Jensen A, Scheve K. Economic Decline, Social Identity, and Authoritarian Values in the United States. International Studies Quarterly. 2022;66(1).

[17] Walter S. The Backlash Against Globalization. Annual Review of Political Science. 2021;24:421-42.

[18] Margalit Y. Costly Jobs: Trade-related Layoffs, Government Compensation, and Voting in U.S. Elections. American Political Science Review. 2011;105(1):166-88.

[19] Rommel T, Walter S. The Electoral Consequences of Offshoring: How the Globalization of Production Shapes Party Preferences. Comparative Political Studies. 2018;51(5):621‚Äì658.

[20] Green DP, Palmquist B, Schickler E. Partisan Hearts and Minds: Political Parties and the Social Identities of Voters. New Haven, CT: Yale University Press; 2002.

[21] Druckman JN, Peterson E, Slothuus R. How Elite Partisan Polarization Affects Public Opinion Formation. American Political Science Review. 2013;107(1):57-79.

[22] Scheve KF, Slaughter MJ. What Determines Individual Trade-Policy Preferences? Journal of International Economics. 2001;54(2):267-92.

[23] Mayda AM, Rodrik D. Why Are Some People (and Countries) More Protectionist than Others? European Economic Review. 2005;49(6):1393-430.

[24] Kojola E. Bringing Back the Mines and a Way of Life: Populism and the Politics of Extraction. Annals of the American Association of Geographers. 2019;109(2):371-81.
[25] Goldstein J, Ballard-Rosa C, Rudra N. Trade as Villain: The Fading American Dream and Declining Support for Globalization. International Political Economy Society Annual Meeting. 2021.
[26] Hemmer C, Katzenstein PJ. Why is There No NATO in Asia? Collective Identity, Regionalism, and the Origins of Multilateralism. International Organization. 2002;56(3):575-607.
[27] Mutz DC. Winners and Losers: The Psychology of Foreign Trade. Princeton University Press, Princeton; 2021.
[28] Barbieri P, Boffelli A, Elia S, Fratocchi L, Kalchschmidt M, Samson D. What can we learn about reshoring after Covid-19? Operations Management Research. 2020;13:131-6.
[29] Acemoglu D, Tahbaz-Salehi A. The Macroeconomics of Supply Chain Disruptions. Review of Economic Studies. 2024;(forthcoming). Available from: https://doi.org/10.1093/restud/rdae038.
[30] Pandya SS. Labor markets and the demand for foreign direct investment. International Organization. 2010;64(3):389-409.
[31] Chilton AS, Milner HV, Tingley D. Reciprocity and Public Opposition to Foreign Direct Investment. British Journal of Political Science. 2017;50(1):129-53.
[32] Zeng K, Li X. Geopolitics, Nationalism, and Foreign Direct Investment: Perceptions of the China Threat and American Public Attitudes toward Chinese FDI. Chinese Journal of International Politics. 2019;12(4):495-518.
[33] Gowa J, Mansfield ED. Power Politics and International Trade. The American Political Science Review. 1993;87(2):408-20.
[34] Mansfield ED, Bronson R. Alliances, Preferential Trading Arrangements, and International Trade. American Political Science Review. 1997;91:94-107.
[35] Davis C, Pratt T. The Forces of Attraction: How Security Interests Shape Membership in Economic Institutions. Review of International Organizations. 2020;Forthcoming.
[36] Carnegie A, Gaikwad N. Public Opinion on Geopolitics and Trade: Theory and Evidence. World Politics. 2022;74(2):167-204.
[37] Schaffer L, Spilker G. Self-interest Versus Sociotropic Considerations: An Information-based Perspective to Understanding Individuals' Trade Preferences. Review of International Political Economy. 2019;26(6):1266-92.
[38] Fordham BO, Kleinberg KB. Trade and Threat Perception. 2008. Unpublished manuscript. Available from: https://ssrn.com/abstract=1144788.
[39] Mansfield ED, Mutz DC. Support for Free Trade: Self-Interest, Sociotropic Politics, and Out-Group Anxiety. International Organization. 2009;63(3):425-57.
[40] Guisinger A. Determining Trade Policy: Do Voters Hold Politicians Accountable? International Organization. 2009;63(3):533-57.
[41] Rho S, Tomz M. Why Don't Trade Preferences Reflect Economic Self-Interest? International Organization. 2017;71(S1):S85-S108.
[42] Casler D, Clark R. Trade Rage: Audience Costs and International Trade. Journal of Conflict Resolution. 2021;65(6):1198-230.
[43] Gallup. How Consumers Felt the Supply Chain Problems; 2021. Accessed: 21-Oct-2024. Available from: https://news.gallup.com/poll/353312/consumers-felt-supply-chain-problems.aspx.

[44] The Wall Street Journal. Car Makers Need More Chips Today, Better Chips Tomorrow; 2021. Accessed: 21-Oct-2024. Available from: https://www.wsj.com/articles/car-makers-need-more-chips-today-better-chips-tomorrow-11626877264.

[45] The Wall Street Journal. Chip Shortage Drives Tech Companies and Car Makers Closer; 2021. Accessed: 21-Oct-2024. Available from: https://www.wsj.com/articles/chip-shortage-drives-tech-companies-and-car-makers-closer-11631455202.

[46] The Wall Street Journal. The Great Car Chip Shortage Will Have Lasting Consequences; 2021. Accessed: 21-Oct-2024. Available from: https://www.wsj.com/articles/the-great-car-chip-shortage-will-have-lasting-consequences-11632737422.

[47] S&P Global. Semiconductor Shortage; 2021. Accessed: 21-Oct-2024. Available from: https://www.spglobal.com/en/research-insights/special-reports/semiconductor-shortage.

[48] U S Department of Commerce. Commerce Semiconductor Data Confirms Urgent Need for Congress to Pass U.S. Innovation and Competition Act; 2022. Accessed: 21-Oct-2024. Available from: https://www.commerce.gov/news/press-releases/2022/01/commerce-semiconductor-data-confirms-urgent-need-congress-pass-us.

[49] Shih W. The Inflation Reduction Act Will Bring Some Manufacturing Back to the U.S.; 2023. Accessed: 21-Oct-2024. Available from: https://www.forbes.com/sites/willyshih/2023/02/22/the-inflation-reduction-act-will-bring-some-manufacturing-back-to-the-u ?sh=2cb77b18b544.

[50] Herrmann RK. How Attachments to the Nation Shape Beliefs About the World: A Theory of Motivated Reasoning. International Organization. 2017 Apr;71(S1):S61-84.

[51] Jardina A. White Identity Politics. Camridge University Press, New York, NY; 2019.

[52] Fiorina MP, Abrams SJ. Political Polarization in the American Public. Annual Review of Political Science. 2008;11:563-88.

[53] Iyengar S, Westwood SJ. Fear and Loathing across Party Lines: New Evidence on Group Polarization. American Journal of Political Science. 2015;59(3):690-707.

[54] Pierson P. Dismantling the Welfare State? Reagan, Thatcher and the Politics of Retrenchment. New York: Cambridge University Press; 1994.

[55] Bartels LM. Unequal Democracy: The Political Economy of the New Gilded Age. Princeton: Princeton University Press; 2016.

[56] International Monetary Fund. Reshoring and the Global Economy; 2024. Accessed: 21-Oct-2024. Available from: https://www.imf.org/-/media/Files/Publications/WP/2024/English/wpiea2024122-print-pdf.ashx.

[57] Hooghe L, Marks G, Lenz T, Bezuijen J, Ceka B, Derderyan S. Review of International Organizations: Toward a Measure of International Authority. The Review of International Organizations. 2019;14:207-38. Accessed: 21-Oct-2024. Available from: https://ideas.repec.org/a/spr/revint/v14y2019i2d10.1007_s11558-019-09353-1.html.

[58] Li X, Zeng K. Individual preferences for FDI in developing countries: experimental evidence from China. Journal of Experimental Political Science. 2017;4(3):195-205.

[59] Vaughn A, Weldzius R. Reshoring Global Supply Chains. 2021. Working paper. Available from: https://bit.ly/3UC45dL.

[60] The New York Times. Semiconductor Boom Faces Worker Shortage in the U.S.; 2023. Ac-

cessed: 21-Oct-2024. Available from: https://www.nytimes.com/2023/05/19/us/politics/semiconductor-worker-shortage.html.

[61] Coppock A, McClellan OA. Validating the Demographic, Political, Psychological, and Experimental Results Obtained from a New Source of Online Survey Respondents. Research and Politics. 2019. Forthcoming.

[62] Kim SE, Margalit Y. Tariffs As Electoral Weapons: The Political Geography of the US-China Trade War. International Organization. 2021;75(1):1-38.

[63] Aronow PM, Kalla J, Orr L, Ternovski J. Evidence of Rising Rates of Inattentiveness on Lucid in 2020. SocArXIV Papers. 2020. Available from: https://osf.io/preprints/socarxiv/8sbe4/.

[64] Peyton K, Huber GA, Coppock A. The Generalizability of Online Experiments Conducted during the COVID-19 Pandemic. SocArXIV Papers. 2020. Available from: https://osf.io/preprints/socarxiv/s45yg/.

[65] Burleigh T, Kennedy R, Clifford S. How to screen out VPS and international respondents using Qualtrics: A protocol. Available at SSRN 3265459. 2018.

[66] Mellon J. Internet Search Data and Issue Salience: The Properties of Google Trends as a Measure of Issue Salience. Journal of Elections, Public Opinion, and Parties. 2014;24(1):45-72.

[67] Faul F, Erdfelder E, Buchner A, Lang AG. Statistical power analyses using G* Power 3.1: Tests for correlation and regression analyses. Behavior research methods. 2009;41(4):1149-60.

[68] Gallup. Consumers Felt Supply Chain Problems. 2021. Available from: https://news.gallup.com/poll/353312/consumers-felt-supply-chain-problems.aspx. Accessed 21 Oct 2024.

[69] Tax Foundation. Trump's Steel and Aluminum Tariffs: Three Years Later. 2024. Available from: https://taxfoundation.org/blog/trump-steel-tariff-aluminum-tariff/. Accessed 21 Oct 2024.
